# In Memoriam: Robert M. Kelly (1953–2026)

**DOI:** 10.1128/aem.00599-26

**Published:** 2026-04-30

**Authors:** Michael W. Adams, Jason M. Haugh, Albert J. Keung, Balaji M. Rao, Sindee L. Simon

**Affiliations:** 1 Department of Biochemistry and Molecular Biology, University of Georgia 174518, Athens, Georgia, USA; 2 Department of Chemical and Biomolecular Engineering, North Carolina State University 242511, Raleigh, North Carolina, USA; 3 Department of Chemical and Biomolecular Engineering, Biotechnology Program—Integrative Sciences Initiative, North Carolina State University 242511, Raleigh, North Carolina, USA; 4 Department of Chemical and Biomolecular Engineering, Golden LEAF Biomanufacturing Training and Education Center, North Carolina State University 242511, Raleigh, North Carolina, USA; Michigan State University, East Lansing, Michigan, USA

**Keywords:** biotechnology, extremophiles

## Abstract

The chemical and biochemical engineering, microbiology, and biotechnology communities deeply mourn the passing of Dr. Robert M. Kelly, the Alcoa Professor of Chemical and Biomolecular Engineering and Director of the Biotechnology Program at NC State University. A towering pioneer in the biology of extremophiles, an esteemed educator and mentor, and a dedicated editor for *Applied and Environmental Microbiology* (AEM), Bob leaves behind an extraordinary legacy that reshaped our understanding of life at high temperatures.

## COMMENTARY

## IN MEMORIAM: ROBERT M. KELLY (1953 –2026)

The chemical and biochemical engineering, microbiology, and biotechnology communities deeply mourn the passing of Dr. Robert M. Kelly, the Alcoa Professor of Chemical and Biomolecular Engineering and Director of the Biotechnology Program at NC State University. Dr. Kelly passed away peacefully on 16 March 2026. A towering pioneer in the biology of extremophiles, an esteemed educator and mentor, and a dedicated editor for *Applied and Environmental Microbiology* (AEM) from 2012 to 2022, Bob leaves behind an extraordinary scientific legacy that fundamentally reshaped our understanding of life at high temperatures.

Over a career spanning more than four decades, Dr. Kelly bridged the gap between fundamental molecular microbiology and applied chemical engineering. He recognized the immense potential of extremophiles long before bioprospecting in extreme environments became commonplace. His research further sought to harness hyperthermophilic archaea and bacteria to solve challenges in bioenergy, green chemistry, and sustainable industrial processes.

To honor his scientific legacy, it is fitting to reflect on the landmark discoveries that defined his career.

## EARLY CAREER AND PIONEERING EXTREMOZYMES FOR INDUSTRIAL BIOCATALYSIS

In the late 1980s and early 1990s, Dr. Kelly was a pioneering contributor to the nascent field of hyperthermophile enzymology. His early work detailed the biological and engineering considerations necessary for cultivating extreme thermophiles (1). Before recombinant methods for producing enzymes from hyperthermophiles were widely established, his laboratory mastered the large-scale cultivation of these microbes, growing archaea like *Pyrococcus furiosus* in continuous cultures at temperatures near 100°C (2, 3). This enabled the native purification of high-temperature biocatalysts for foundational characterization studies (4, 5).

Dr. Kelly was instrumental in bringing "extremozymes" to the forefront of industrial biocatalysis. His research strategically selected hyperthermophilic esterases and lipases for specialized industrial applications, including the racemic resolution of 2-aryl propionic esters (6). Operating extensively within pure biochemical characterizations during this era, his group also elucidated the functional and structural stability of the archaeal 20S proteasome, utilizing it as a simple, thermostable model system for core particle function (7, 8).

## PHYSIOLOGY, GENOMICS, AND STRESS RESPONSE OF EXTREMOPHILES

As molecular tools advanced into the 2000s, Dr. Kelly’s laboratory became a driving force in establishing extreme thermophiles as model genetic and physiological systems. His group conducted exhaustive transcriptional analyses of dynamic heat shock responses and functional genomics in the hyperthermophilic bacterium *Thermotoga maritima* (9, 10). Concurrently, his research investigated the genome plasticity and metabolic adjustments of the thermoacidophilic archaeon *Sulfolobus solfataricus* in response to thermal and pH stress, bridging the gap between genomic potential and environmental adaptation (11).

## ENGINEERING HYPERTHERMOPHILES WITH NOVEL ENZYMES FOR FUELS AND CHEMICALS

A hallmark of Dr. Kelly’s career was his highly productive, 35-year collaboration with Dr. Michael W. W. Adams, resulting in over 70 joint peer-reviewed publications since 2013 alone. Together, they pioneered the engineering of hyperthermophilic microbes to produce useful fuels and chemicals, an endeavor that fundamentally expanded the utility of extreme thermophiles as metabolic engineering platforms (12–17).

Dr. Kelly’s group dissected the reaction kinetics governing the 3-hydroxypropionate/4-hydroxybutyrate CO_2_ fixation cycle in extremely thermoacidophilic archaea like *Metallosphaera sedula*, laying the groundwork for microbial electrofuels (12, 18, 19). They developed genetic tools to insert synthetic biochemical pathways, manipulating fermentation in *Pyrococcus furiosus* to drive the biological conversion of carbon dioxide and hydrogen gas into liquid fuels (20, 21). Through ingenious engineering, including expressing bacterial bifunctional alcohol dehydrogenases, his team achieved ethanol and 1-propanol production up to 95°C (22–24). Recent efforts successfully engineered ethanol and acetone production, guided by deep structural and kinetic characterizations of essential pathway enzymes (25, 26).

## DECONSTRUCTION OF PLANT BIOMASS BY NEW SYNTHETIC ENZYME PATHWAYS

In the 2010s and 2020s, Dr. Kelly revolutionized our understanding of lignocellulose deconstruction through his comprehensive investigation of the genus *Caldicellulosiruptor*, affectionately known as 'Caldi World' (27). Through comparative, core, and pan-genomic analyses (28–31), his team discovered that *Caldicellulosiruptor* species degrade cellulose via a suite of novel, multidomain extracellular glycosyl hydrolases and specialized cellulose-binding proteins (täpirins) rather than conventional cellulosomes (32–36). He elaborated on the transcriptional regulation of these systems and engineered them to improve biomass solubilization (37–42).

His group conducted groundbreaking quantitative fermentations of unpretreated, transgenic poplar and switchgrass, proving that lowering lignin content drastically improves bioavailability for thermophilic conversion (43–46). By engineering these strains to produce ethanol and higher alcohols (47, 48), he established the viability of industrial scale-up of thermophilic biomass conversion. His work also revealed an added benefit of how extreme thermophilic processing circumvents the microbial contamination common in lower-temperature bioenergy workflows (49).

## BIOLEACHING AND SULFUR BIOOXIDATION

In parallel with his work in biomass and synthetic biology, Dr. Kelly made profound, continual contributions throughout his career to the understanding of heavy metal biooxidation and biomining. Beginning with early studies on culturing conditions for *Sulfolobus acidocaldarius* in hyperbaric bioreactors (50), his research explored sulfur and metal chemistries. In particular, he investigated the genomics and toxin-antitoxin systems, such as VapC6 and VapB, that regulate thermophilicity and biofilm dispersal in the archaeal family *Sulfolobaceae* (51–53). In his most recent work, his laboratory traced the ancestral trajectory of sulfur oxidation in thermoacidophiles and provided critical insights into the regulation and efficacy of bioleaching (54–56).

## LEGACY AND HONORS

Dr. Kelly was a driving force in establishing species from the genera *Thermotoga*, *Caldicellulosiruptor*, *Metallosphaera*, *Pyrococcus*, and *Sulfolobus* as model extremophile systems. Beyond his pioneering and prolific research, he co-edited three volumes of *Methods in Enzymology* on hyperthermophilic enzymes (57–59) and helped organize seminal gatherings, including the 1999 Enzyme Engineering Conference. In 2018, the American Society for Microbiology honored his transformative career with the DuPont Biosciences Award in Applied and Environmental Microbiology. Dr. Kelly was elected a fellow of AIMBE (1996), AAAS (2007), and AIChE (2024). He was also the recipient of the 2022 Amgen Biochemical and Molecular Engineering Award, the NC State College of Engineering RJ Reynolds Award for Excellence in Teaching, Research and Extension (2002), the Lifetime Achievement Award from the International Society for Extremophiles (2018), and the Charles D. Scott Award from Society for Industrial Microbiology and Biotechnology (2025). He received the Alexander Quarles Holladay Medal for Excellence, the highest honor for faculty at NC State University in 2021.

His leadership extended to his home institution, where he directed the NC State Biotechnology Program for 25 years and led NIH T32 and DOE GAANN Pre-Doctoral Training Programs for over two decades. He was widely known as a kind and supportive mentor to hundreds of trainees in molecular biotechnology, to junior faculty, and to colleagues. He often stated to his colleagues that his work with students through the BIT Program was the most meaningful of his career.

Dr. Kelly’s ingenuity, innovativeness, and remarkable productivity brought the world of extremophile enzymology from fundamental discovery to biotechnological application. His pedagogical vision and care will endure through his profound contributions to the generation of scientists he mentored. He will be deeply missed.

Dr. Kelly is survived by his wife, Maureen Kelly; his children, Elizabeth Kelly, Caitlin Kelly Gaylord (Ryan), and Michael Kelly (Erica); his grandchildren, Jack, Madden, and Bennett; his brothers, James Kelly and Thomas Kelly; and numerous cousins, nieces, and nephews. He is preceded in death by his parents, Robert M. Kelly and Audrey A. Kelly. The Bob Kelly Biotechnology Endowment has been established by the family in memory of Bob. The fund will aid the NC State BIT program that he directed for 25 years: https://biotech.ncsu.edu/.
